# Policy Making in Newborn Screening Needs a Structured and Transparent Approach

**DOI:** 10.3389/fpubh.2017.00053

**Published:** 2017-03-21

**Authors:** Marleen E. Jansen, Karla J. Lister, Henk J. van Kranen, Martina C. Cornel

**Affiliations:** ^1^Section Community Genetics, Department of Clinical Genetics, Amsterdam Public Health Research Institute, Amsterdam, Netherlands; ^2^Institute for Public Health Genomics, School for Oncology and Developmental Biology (GROW), Faculty of Health, Medicine, and Life Sciences, Maastricht University, Maastricht, Netherlands; ^3^Screening Policy Section, Office of Population Health Genomics, Department of Health, Government of Western Australia, Perth, WA, Australia; ^4^Center for Nutrition, Prevention and Health Services, National Institute for Public Health and the Environment, Bilthoven, Netherlands

**Keywords:** decision making, newborn screening, public health policy, genomic screening, genetic testing

## Abstract

**Purpose:**

Newborn bloodspot screening (NBS) programs have expanded significantly in the past years and are expected to expand further with the emergence of genetic technologies. Historically, NBS expansion has often occurred following *ad hoc* consideration of conditions, instead of a structured and transparent approach. In this review, we explore issues pertinent to NBS policy making, through the lens of the policy cycle: (a) agenda setting, (b) policy advice, (c) policy decision, (d) implementation, and (e) evaluation.

**Methods:**

A literature search was conducted to gather information on the elements specific to NBS and its policy making process.

**Results:**

The review highlighted two approaches to nominate a condition: a structured approach through horizon scanning; and an *ad hoc* process. For assessment of a condition, there was unanimous support for a robust process based on criteria. While the need to assess harms and benefits was a repeated theme in the articles, there is no agreed-upon threshold for benefit in decision-making. Furthermore, the literature was consistent in its recommendation for an overarching, independent, multidisciplinary group providing recommendations to government. An implementation plan focusing on the different levels on which NBS operates and the information needed on each level is essential for successful implementation. Continuously monitoring, and improving a program is vital, particularly following the implementation of screening for a new condition. An advisory committee could advise on implementation, development, review, modification, and cessation of (parts of) NBS.

**Conclusion:**

The results highlight that there are a wave of issues facing NBS programs that policy makers must take into account when developing policy processes. What conditions to screen, and the technologies used in NBS, are both up for debate.

## Introduction

Newborn bloodspot screening (NBS) is the longest running and most successful population screening program worldwide (1). NBS tests newborns within the first days of life for multiple serious conditions (2). The traditional aim of NBS is to prevent serious consequences for the newborn by enabling timely diagnoses and treatment for early onset childhood conditions (3). In recent years, technological developments, changes in understanding of conditions, and new treatments, have fueled the expansion of NBS (4, 5). The aim of this study is to explore issues influencing each phase of the policy cycle. In doing so, this study provides policy makers insight in the pressures facing NBS to inform them on approaches to successfully guide programs into the future.

An archetypal example of the policy pressures facing NBS was the advent of tandem mass spectrometry (MS/MS), and the resultant impact it had upon programs worldwide. This technology emerged in the 1990s and made it possible to test for several conditions at once in a cost-effective manner (4, 5). Correspondingly, several programs adopted the technology and significantly increased the number of conditions screened. Programs using these technologies regularly screen from 9 to over 50 conditions (1, 6–9). Even more than MS/MS, genetic technologies may enable screening for a larger number of conditions (3, 10, 11). Debate abounds in the academic literature on the appropriateness of expanding NBS (4, 10, 12): some authors advocate for targeted approaches (13, 14); while elsewhere next generation sequencing is being applied in the research setting to study it’s potential for NBS (15, 16).

Previous expansions and the divergent programs that have evolved, suggest that the emergence of genetic technologies is likely to be a significant turning point for NBS. Given the reach of NBS, that NBS tests our most vulnerable population, and the potential to increasingly expand programs, it is essential that decisions on what to screen are carefully considered. Thus policy approaches are needed which can successfully navigate in the changing environment (14). It can be expected that an expansion occurring in some countries will become an example of what is possible for other countries (17). At a minimum, further debate will emerge on the pros and cons of expanded screening, and experts, consumers, and advocacy groups are likely to increase calls for screening for specific conditions (17). The emergence of debates on further expanding NBS presents decision makers worldwide with the challenge of weighing the benefits and harms of screening in the changing landscape of NBS. Policy frameworks, which are developed in light of the range of policy issues, will be essential for policy makers to ensure their programs can effectively respond to the pressures facing the program, now and in the future.

Given the pressures for NBS, the current study aims to identify what the scientific literature outlines are the key policy considerations currently facing NBS. This is achieved by exploring issues pertinent to NBS policy making, through the lens of the policy cycle (10): (a) agenda setting, (b) policy advice, (c) policy decision, (d) implementation, and (e) evaluation. Without detailing current developments in genomic technologies, we aim to explore issues influencing each phase of the policy cycle. In doing so, this study will enable policy makers responsible for existing or emerging NBS programs to consider the best policy structure to respond to the changing environment in which NBS operates.

## Methods

We explored academic literature to summarize relevant factors pertinent to policy making for NBS. International policy making processes have been recently reviewed elsewhere (1, 12). The current study builds upon what is known about the tangible policy making process, by highlighting issues facing NBS identified in academic literature. This then provides policy makers an outline of the issues that should be considered in the development of policies. To be included in the review an article needed to discuss one or more of the following topics: nomination of a condition (agenda setting); consideration of a condition (policy advice); deciding on a condition (policy decision); addition of a condition (implementation); or quality assurance and improvement (evaluation). Articles were also included if they discussed all elements of the policy cycle, such as mention of a comprehensive policy framework for NBS policy making.

We searched PubMed for articles regarding *newborn screening* and *policy*. We combined the two key search terms with the following search terms: 1. *program development*, 2. *decision-making*, 3. *governance*, 4. *management*, 5. *perspective*, 6. *future*, and 7. *disease* or *condition*. Only English publications on dried bloodspot screening were included. Articles concerning other types of newborn screening (e.g., hearing, hip dysplasia) were excluded, because we wanted to focus on the complex policy making specific to bloodspot screening.

## Results

The initial literature search identified 59 articles. Twenty-seven articles discussed one or more of the elements of the policy cycle (Table S1 in Supplementary Material). Most literature originated from western societies, predominantly the USA (13 of 27 articles, Table S1 in Supplementary Material) and was initiated from a clinical background rather than a public health background (Figure S1 in Supplementary Material). The main technology discussed in the articles shows a shift through time from MS/MS toward discussing genetic technologies as the challenge for NBS: 3 of 13 articles until 2008 discuss mainly genetic technologies and 6 of 14 published since 2009 (Table S1 in Supplementary Material). Recent articles discussing MS/MS report on results from current screening programs or previous decision-making processes (18). The following outlines the results, stepping through the policy cycle.

### Agenda Setting: Nominating a Condition

The review highlighted two approaches to nominating a condition. The first is a structured approach focused on horizon scanning; the second approach is much more *ad hoc* and influenced by external drivers, such as advocacy (18–20).

The structured, horizon scanning approach generally includes an independent body that undertakes horizon scanning to identify a range of relevant conditions to evaluate for NBS and support expansion through an evidence-based process (10, 21, 22). Such an approach has been successfully used by several countries in agenda setting based on an objective threshold of criteria (18, 22). In horizon scanning, potential conditions are identified and recommended for further in-depth review, through initial assessment of criteria. Contrary to the organized horizon scanning approach, the majority of the literature focused on an *ad hoc* approach. In this approach, conditions became the focus of an assessment for NBS in response to new technologies, broader disease definition, insight into pathophysiology, and advocacy (5, 23–26). In the past, NBS policy direction and program expansion have been strongly influenced by technological drivers, often evaluated *ad hoc* (18–20).

Advocacy is a key driver for change within NBS. Pressure by consumers, clinicians, and scientists to screen for a condition dates back to the very first condition screened in NBS, phenylketonuria (PKU). NBS for PKU was advocated by Dr Guthrie, whose son was born mentally handicapped and whose niece experienced intellectual disability due to undiagnosed and unmanaged PKU (5). Dr Guthrie developed a test to identify the condition and advocated for mass screening of PKU through community support groups (5). Recent examples of advocate pressure leading to the introduction of a condition include in the instance of X-linked adrenoleukodystrophy and Krabbe disease in the USA (5, 10, 27). However, the benefits of screening, for Krabbe are disputed in literature and referred to as “dangerous and expensive” (27).

### Policy Advice: Assessment of a Condition

Within the literature reviewed, there was unanimous support for a robust assessment process based on criteria. However, a key issue relating to the assessment of nominated conditions centered on the appropriateness of using criteria originating from the Wilson and Jungner principles (10, 28, 29). Criticisms are voiced that the Wilson and Jungner principles are developed to evaluate individual conditions, while modern day technology pushes toward the possibility and sometimes the need to evaluate groups of conditions at once (30, 31). Furthermore, there is no objective tool developed from the Wilson and Jungner principles, which leaves them open to interpretation into different criteria between programs (31).

While the need to assess harms and benefits was a repeated theme in the articles, there is no agreed-upon threshold for benefit (10, 22, 29). This is essential to effectively explore the benefits and harms of screening, to ensure that the former outweighs the latter. The benefit of screening is intrinsically related to the primary aim of screening, which is predominantly to avoid preventable harm in newborns. The aim and beneficiary screening should both be specified in policies, as they are open to interpretation (9, 32, 33). That is, to support assessing the appropriateness of a condition, there needs to a clear understanding of who will benefit from screening, what is the perceived benefit, and how should it be weighed in decisions (10, 22). In general, three groups were mentioned as potential beneficiaries of NBS: the child, the family, and/or society (34). In the recent report from the Health Council of the Netherlands, the beneficiary of screening was specifically defined as the child. Consequently, this lead to conditions without clear clinical benefits to the child to be assessed as inappropriate for inclusion within NBS (12).

### Policy Decision: Deciding on a Condition

The literature focused on two key areas when deciding whether to screen a condition. The first focused on who makes the decision, and the second focused on the evidence on which the decision is made. The literature was consistent in its recommendation for an overarching, independent, multidisciplinary group providing recommendations to government (18, 24, 28).

In terms of evidence, authors of the reviewed literature identified that decisions in NBS often need to be made based on incomplete information (22). A main concern identified in several articles, is the lack of data to support evidence-based decisions. There is the need for interoperable databases to collect sufficient data on the diseases considered and included in NBS (10, 23, 35). Alternatively, authors suggested that innovations in NBS should be implemented in a research paradigm, to facilitate data collection for policy decisions, and gain informed consent from parents participating with their child(ren) in the study (22, 30, 33, 35). Pilot studies are vital to the development of a strong evidence base to support decision-making regarding the addition of new conditions. As shown in Denmark, the Faroe Island, and Greenland, a pilot program of 7 years eventually provided information for evaluation and the subsequent decision to not include 11 conditions in the routine screening program in 2009 (36).

### Implementation: Addition of a Condition

Once a condition is approved for implementation in a NBS program, an implementation plan focusing on the different levels on which NBS operates and the information needed at each level should be developed (21, 37, 38). Issues across programs are similar when looking at implementation and relate to five fields: education, finances, logistics, politics, and culture (5, 24). These fields extend beyond “public health” to also include follow-up in the clinical setting. Key issues include the need for work flow across these fields to be coordinated, and ensuring professionals have the relevant skills and knowledge for the new condition(s) (24, 39). Issues to ensure skills and knowledge are particularly challenging where conditions are being identified in the pre-symptomatic phase, and there may be a lack of evidence or consensus in clinical guidelines. This will be further challenged if the preferred technology moves toward genome-based technologies, which can identify genetic variations that have implications for family members. These technologies will lead to issues relating to privacy and confidentiality, residual specimen storage and usage policies, and educational material to become even more pressing (40).

### Evaluation: Quality Assurance and Improvement

Continuously monitoring, and improving a program is vital, particularly following the implementation of screening for a new condition (30). It is possible that a condition assessed as being appropriate to screen, will not meet the parameters upon which this decision was based. For example, the false positives or negatives recorded for a test within a trial period might not align with those that occur in the real world setting. Thus quality assurance (QA) is required to monitor the program’s performance against defined targets to ensure it aligns with the anticipated outcomes (22, 29, 34).

QA provides essential ongoing assessment of feasibility, cost, and equitable delivery of testing (10, 32, 37). Some authors suggest principles for QA, such as clear guidelines on responsibilities throughout the chain of NBS; standards on aspects regarding confidentiality; and protocols for storage of blood spot specimens (21). Importantly, QA should be complemented by quality improvement (QI). QI builds upon QA, to drive improvements and achieve success. Issues for QI within NBS include managing the improvement process across the NBS system: from health-care professionals to laboratory experts. Ways to overcome fragmentation of providing information on the key indicators while gaining data on them from all parts of NBS can be providing training, develop written educational materials for parents and health-care professionals, and redesign laboratory slips for blood collection (21).

### Policy Cycle in General

Authors advocated a transparent, structured, and evidence-based process (22, 25, 41). Policy making for NBS can be governed both locally and nationally (38, 42). Governance is a process that focuses on balancing competing influences and demands (43). The need for harmonization of national policies is often referred to in literature: to ensure a national balance in competing interests and equity in access to early interventions (29, 31). A central body like a national or federal government should play a core role in overseeing NBS. In addition, consultation and engagement is a key theme, which some authors highlight should be managed through a multidisciplinary advisory committee providing advice through the policy cycle (28). An advisory committee could advise them on implementation, development, review, modification, and cessation of (parts of) NBS (21, 29, 38).

## Discussion

Stepping through the policy cycle illustrated that NBS is on the precipice of great change. Programs are facing a wave of pressures, including in response to new treatments and new technologies. The history of NBS suggests the programs are flexible in responding to a continually changing environment. However, the historical *ad hoc* approach to adding conditions to NBS is recognized as potentially problematic in the light of future developments. Current NBS programs might contain conditions that have not been robustly evaluated through an agreed policy advice process. Future developments and challenges highlight that policy makers need to take stock of the issues facing the programs, and develop policies that will ensure safe and appropriate growth of programs (22, 25, 41).

The growing number of conditions that could be screened is a key issue for NBS programs, particularly in the face of pressures from next generation sequencing (13, 44). Moreover, the potentially growing number of conditions extends beyond what is technologically possible, to challenge the fundamental purpose of the programs. Internationally, there are increasing calls to move further beyond the traditional aim of NBS, and screen for “untreatable” conditions (33, 34). Untreatable conditions do not always have a certain treatment benefit or treatment is not urgently needed in the newborn period. For untreatable conditions to be implemented in NBS, some argue that the aim has to shift from clinical benefits solely for the child, to include family benefits (24, 29). Such a shift in the focus of beneficiary beyond the newborn, will lead to a vast increase in the number and type of conditions eligible for screening (3, 22): a great number of conditions may have family benefit through information on relevant reproductive options compared to a limited amount of conditions that have direct clinical benefit for the newborn (34).

The above issues overwhelmingly outline the need for robust and considered policy making for NBS. However, it is unclear whether such policy making can fully combat the pressures facing the program. Many nomination processes can still be considered passive where a nomination is awaited, instead of active horizon scanning for relevant conditions. Further, should there be a shift in focus or a push for more conditions to be screened, it is recommended that this be accompanied with consideration as to whether NBS is the right place to screen for such conditions. Specifically, in order to protect the programs and ensure they stay true to their aim, consideration should be given to preconception, prenatal, or screening during early childhood.

Our study has several limitations, data from the USA were overrepresented (13 of the 27 articles) and a sample of 27 articles might not be representative for international policy making in NBS. Further, the policy cycle is theoretical. As such, the recommendations from academic literature are prone to interpretational disparity between theory and practice. Nonetheless, this review shows relevant aspects in policy making and addresses gaps in the current processes. Our results suggests that there is the need for a structured and timely approach that responds to the changing environment.

Through a systematic, continuous policy process, NBS programs will be able to anticipate developments, as opposed to being reactive and heavily influenced by external drivers. A policy process that is developed in light of the issues raised here will help the programs to anticipate challenges and progress in a safe and effective way. A framework to facilitate this approach should be strived for. Only by making careful and considered decisions, can we ensure that NBS of the future is as successful as the existing programs we know today.

## Author Contributions

MJ contributed to the conception and design of the work, analysis and interpretation of data, drafted the work, and revised it. KL contributed to the interpretation of data and critically revised the work. HK contributed to the design of the work and critically revised it. MC contributed to the conception and design of the work, interpretation of data, and critically revised the work. All authors approved the version to be published, and agreed to be accountable for all aspects of the work in ensuring that questions related to the accuracy or integrity of any part of the work are appropriately investigated and resolved.

## Conflict of Interest Statement

The authors declare that the research was conducted in the absence of any commercial or financial relationships that could be construed as a potential conflict of interest.

## Supplementary Material

Figure S1 **Proportion of the affiliations of the first authors of the articles included in the review**.

Table S1 **Articles included in the review (*n* = 26) with a short summary of each article, as well as the main stakeholder groups discussed, articles are sorted based on publication date to illustrate the shift in the technologies discussed**.

## References

[1] Therrell BL Padilla CD Loeber JG Kneisser I Saadallah A Borrajo GJC Current status of newborn screening worldwide: 2015. Semin Perinatol (2015) 39:171–87. 10.1053/j.semperi.2015.03.002 25979780

[2] Health Council of the Netherlands. Neonatal Screening: New Recommendations. The Hague: Health Council of the Netherlands (2015).

[3] Bombard Y Miller FA Hayeems RZ Avard D Knoppers BM. Reconsidering reproductive benefit through newborn screening: a systematic review of guidelines on preconception, prenatal and newborn screening. Eur J Hum Genet (2010) 18:751–60. 10.1038/ejhg.2010.13 20197792 PMC2987364

[4] Moyer VA Calonge N Teutsch SM Botkin JR Expanding newborn screening: process, policy, and priorities. Hastings Cent Rep (2008) 38(3):32–9. 10.1353/hcr.0.0011 18581935

[5] Therrell BL. U.S. newborn screening policy dilemmas for the twenty-first century. Mol Genet Metab (2001) 74:64–74. 10.1006/mgme.2001.3238 11592804

[6] Dezateux C. Evaluating newborn screening programmes based on dried blood spots: future challenges. Br Med Bull (1998) 54(4):877–90. 10.1093/oxfordjournals.bmb.a011735 10367420

[7] Pollitt RJ. Introducing new screens: why are we all doing different things? J Inherit Metab Dis (2007) 360:423–9. 10.1007/s10545-007-0647-2 17616846

[8] Pollitt RJ. Newborn blood spot screening: new opportunities, old problems. J Inherit Metab Dis (2009) 32:395–9. 10.1007/s10545-009-9962-0 19412659

[9] Potter BK Avard D Wilson BJ. Newborn blood spot screening in four countries: stakeholder involvement. J Public Health Policy (2008) 29(1):121–42. 10.1057/palgrave.jphp.3200161 18368024

[10] Bailey DB Beskow LM Davis AM Skinner D. Changing perspectives on the benefits of newborn screening. Ment Retard Dev Disabil Res Rev (2006) 12:270–9. 10.1002/mrdd.20119 17183569

[11] Topol EJ. Individualized medicine from prewomb to tomb. Cell (2014) 157(1):241–53. 10.1016/j.cell.2014.02.012 24679539 PMC3995127

[12] Jansen ME Metternick-Jones SC Lister KJ International differences in the evaluation of conditions for newborn bloodspot screening: a review of scientific literature and policy documents. Eur J Hum Genet (2016) 25(1):10–6. 10.1038/ejhg.2016.126 27848945 PMC5159762

[13] Howard HC Knoppers BM Cornel MC Wright Clayton E Senecal K Borry P Whole-genome sequencing in newborn screening? A statement on the continued importance of targeted approaches in newborn screening programmes. Eur J Hum Genet (2015) 23(12):1593–600. 10.1038/ejhg.2014.289 25626707 PMC4795188

[14] Knoppers BM Sénécal K Borry P Avard D Whole-genome sequencing in newborn screening programs. Sci Transl Med (2014) 6(229):229cm2 10.1126/scitranslmed.3008494 24670681

[15] Roberts JS Dolinoy DC Tarini BA. Emerging issues in public health genomics. Annu Rev Genomics Hum Genet (2014) 15:461–80. 10.1146/annurev-genom-090413-025514 25184533 PMC4229014

[16] Nowogrodzki A Should Babies Have Their Genomes Sequenced? (2015). Available from: http://www.technologyreview.com/news/538931/should-babies-have-their-genomes-sequenced/

[17] Metternick-Jones SC Lister KJ Dawkins HJS White CA Weeramanthri TS. Review of current international decision-making processes for newborn screening: lessons for Australia. Front Public Health (2015) 3:214. 10.3389/fpubh.2015.00214 26442241 PMC4564656

[18] Fischer KE Grosse SD Rogowski WH. The role of health technology assessment in coverage decisions on newborn screening. Int J Technol Assess Health Care (2011) 27(4):313–21. 10.1017/S0266462311000468 22004771

[19] Avard D Stanton Jean M Grégoire G Page M Public involvement in health genomics: the reality behind the policies. Int J Consum Stud (2010) 34(5):508–24. 10.1111/j.1470-6431.2010.00914.x

[20] EURORDIS. The Voice of Rare Disease Patients in Europe. (2015). Available from: http://www.eurordis.org/

[21] Atkinson K Zuckerman B Sharfstein JM Levin D Blatt RJR Koh HK A public health response to emerging technology: expansion of the Massachusetts newborn screening program. Public Health Rep (2001) 116:122–31. 10.1016/S0033-3549(04)50004-3 11847298 PMC1497306

[22] Grosse SD Boyle CA Kenneson A Khoury MJ Wilfond BS From public health emergency to public health service, the implications of evolving criteria for newborn screening panels. Pediatrics (2006) 117(3):923–9. 10.1542/peds.2005-0553 16510675

[23] Bailey DB Skinner D Davis AM Withmarsh I Powell C. Ethical, legal, and social concerns about expanded newborn screening: fragile X syndrome as a prototype for emerging issues. Pediatrics (2008) 121:e693–704. 10.1542/peds.2007-0820 18310190

[24] Dhondt J. Expanded newborn screening: social and ethical issues. J Inherit Metab Dis (2010) 33(Suppl 2):S211–7. 10.1007/s10545-010-9138-y 20544288

[25] Hayeems RZ Miller FA Bombard Y Avard D Carroll J Wilson B Expectations and values about expanded newborn screening: a public engagement study. Health Expect (2013) 18(3):419–29. 10.1111/hex.12047 23369110 PMC5060787

[26] Therrell BL Jr. Considerations in choosing screening conditions: one (US) approach. Ann Acad Med Singapore (2008) 37(12 Suppl):22–5. 19904477

[27] Dees RH Kwon JM The ethics of Krabbe newborn screening. Public Health Ethics (2013) 6(1):114–28. 10.1093/phe/phs033

[28] Plass AMC van El CG Pieters T Cornel MC Neonatal screening for treatable and untreatable disorders: prospective parents’ opinions. Pediatrics (2009) 125:e99–106. 10.1542/peds.2009-0269 20026497

[29] Elliman DAC Dezateux C Bedford HE Newborn and childhood screening programmes: criteria, evidence, and current policy. Arch Dis Child (2002) 87:6–9. 10.1136/adc.87.1.6 12089109 PMC1751146

[30] Arn PH. Newborn screening: current status. Health Aff (2007) 26(2):559–66. 10.1377/hlthaff.26.2.559 17339686

[31] Petros M Revisiting the Wilson-Jungner criteria: how can supplemental criteria guide public health in the era of genetic testing? Genet Med (2012) 14(1):129–34. 10.1038/gim.0b013e31823331d0 22237442

[32] Burke W Laberge A-M Press N. Debating clinical utility. Public Health Genomics (2010) 13:215–23. 10.1159/000279623 20395690 PMC2865394

[33] Clayton EW What should be the role of public health in newborn screening and prenatal diagnosis? Am J Prev Med (1999) 16(2):111–5. 10.1016/S0749-3797(98)00142-1 10343887

[34] Bailey DB. The blurred distinction between treatable and untreatable conditions in newborn screening. Health Matrix (2009) 19(1):141–53. 19459541

[35] Botkin JR Lewis MH Watson MS Swoboda KJ Anderson R Berry SA Parental permission for pilot newborn screening research: guidelines from the NBSTRN. Pediatrics (2014) 133:e410–7. 10.1542/peds.2013-2271 24394680 PMC3904278

[36] Lund AM Hougaard DM Simonsen H Andresen BS Christensen M Dunø M Biochemical screening of 504,049 newborns in Denmark, the Faroe Islands and Greenland—experience and development of a routine program for expanded newborn screening. Mol Genet Metab (2012) 107(3):281–93. 10.1016/j.ymgme.2012.06.006 22795865

[37] Comeau AM Parad R Gerstle R O’Sullivan BP Dorkin HL Dovey M Challenges in implementing a successful newborn cystic fibrosis screening program. J Pediatr (2005) 147:S89–93. 10.1016/j.jpeds.2005.08.006 16202791

[38] Cornejo V Raimann E Cabello JF Valiente A Becerra C Opazo M Past, present and future of newborn screening in Chile. J Inherit Metab Dis (2010) 33(Suppl 3):S301–6. 10.1007/s10545-010-9165-8 20683669

[39] Kasper DC Ratschmann R Metz TF Mechtler TP Moslinger D Konstantopoulou V The national Austrian newborn screening program – eight years experience with mass spectrometry. Past, present, and future goals. Wien Klin Wochenschr (2010) 122:607–13. 10.1007/s00508-010-1457-3 20938748

[40] Bombard Y Miller FA Hayeems RZ Barg C Cressman C Carroll JC Public views on participating in newborn screening using genome sequencing. Eur J Hum Genet (2014) 22(11):1248–54. 10.1038/ejhg.2014.22 24549052 PMC4200434

[41] Benkendorf J Goodspeed T Watson MS. Newborn screening residual dried blood spot use for newborn screening quality improvement. Genet Med (2010) 12:S269–72. 10.1097/GIM.0b013e3181fea489 21150375

[42] Hiller EH Landenburger G Natowicz MR Public participation in medical policy-making and the status of consumer autonomy: the example of newborn screening programs in the United States. Am J Public Health (1997) 87(8):1280–8. 10.2105/AJPH.87.8.1280 9279262 PMC1381087

[43] WHO. Governance: World Health Organization. (2015). Available from: http://www.who.int/healthsystems/topics/stewardship/en/

[44] Chen R Shi L Hakenberg J Naughton B Sklar P Zhang J Analysis of 589,306 genomes identifies individuals resilient to severe Mendelian childhood diseases. Nat Biotechnol (2016) 34(5):531–8. 10.1038/nbt.3514 27065010

