# Supplementary figures and images for: Policy Making in Newborn Screening Needs a Structured and Transparent Approach
Source: Front Public Health. 2017 Mar 21;5:53. doi: 10.3389/fpubh.2017.00053 (PMC5359248; doi:10.3389/fpubh.2017.00053)

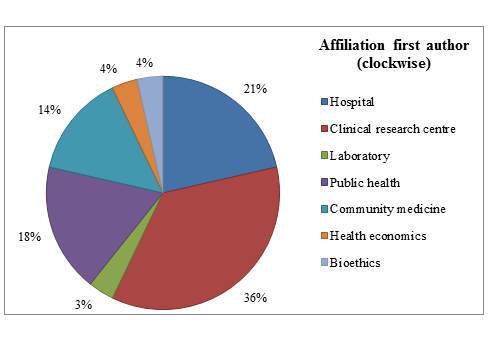

Supplement: Figure S1 — Proportion of the affiliations of the first authors of the articles included in the review. [file Image_1.tif]
