# Supplementary material for: Policy Making in Newborn Screening Needs a Structured and Transparent Approach
Source: Front Public Health. 2017 Mar 21;5:53. doi: 10.3389/fpubh.2017.00053 (PMC5359248; doi:10.3389/fpubh.2017.00053)
Supplement: Table S1 — Articles included in the review (n = 26) with a short summary of each article, as well as the main stakeholder groups discussed, articles are sorted based on publication date to illustrate the shift in the technologies discussed. [file Table_1.docx]

Supplementary Material

Policy-making in newborn screening needs a transparent and structured approach

ME Jansen, KJ Lister, HJ van Kranen, MC Cornel

*** Correspondence:** Marleen Jansen, [m.jansen5@vumc.nl](mailto:m.jansen5@vumc.nl)

**Supplementary Table 1. Articles included in the review (n = 26) with a short summary of each article, as well as the main stakeholder groups discussed, articles are sorted based on publication date to illustrate the shift in the technologies discussed.**

| **Author**  **(year of publication)** | **Main technology discussed (MS/MS; genetic technologies)** | **Key words** | **Type of article, region** |
| --- | --- | --- | --- |
| Hiller *et al*. (1997)(1) | PKU and CH | N/A | Research, USA |
| Atkinson *et al*. (2001)(2) | MS/MS | N/A | Practice article, USA |
| Therrell (2001)(3) | MS/MS and genetic technologies | newborn screening; policy; tandem mass spectrometry; DNA; public health. | Review, USA |
| Elliman *et al*. (2002)(4) | MS/MS | N/A | Review, UK |
| Comeau *et al*. (2005)(5) | Genetic technologies | N/A | Review, USA |
| Bailey *et al*. (2006)(6) | MS/MS and genetic technologies | Consumer advocacy; early intervention; family; infant; newborn; newborn screening | Review, USA |
| Grosse *et al*. (2006)(7) | MS/MS and genetic technologies | N/A | Review, USA |
| Arn (2007)(8) | MS/MS and genetic technologies | N/A | Review, USA |
| Borowski *et al*. (2007)(9) | None | Health policy, Healthcare funding, Health technology assessment, Decision making, Organizational | Research, USA |
| Bailey *et al*. (2008)(10) | Genetic technologies | Newborn screening | Review, USA |
| Little *et al*. (2008)(11) | Genetic technologies | Biobanking; Birth defects surveillance; Canada; Genetic counseling; Genetic testing; Health technology assessment; Newborn screening; Prenatal diagnosis | Review, Canada |
| Potter *et al*. (2008)(12) | MS/MS and genetic technologies | neonatal screening, policy development, stakeholder involvement | Review, UK,USA, Australia, Canada |
| Therrell (2008)(13) | MS/MS and genetic technologies | Newborn screening, Paediatrics, Screening | Report, USA |
| Bailey (2009)(14) | MS/MS and genetic technologies | N/A | Review, USA |
| Plass *et al*. (2009)(15) | Genetic technologies | neonatal screening, extension, parents’ opinion, untreatable diseases, childhood onset diseases | Research, NL |
| Potter *et al*. (2009)(12) | Genetic technologies | Ethical, legal, and social issues (ELSIs); Genetic screening; Health technology assessment; Neonatal screening; Prenatal screening; Public health ethics | Workshop report, Canada |
| Simopoulous (2009)(16) | Genetic technologies | Ethical, legal, social, and economic aspects; Genetic counseling; Genetics education; Genetic screening; Public acceptance | Review, NA |
| Benkendorf *et al*. (2010)(17) | MS/MS and genetic technologies | newborn screening; dried blood spots | Meeting report, USA |
| Bombard *et al*. (2010)(18) | Genetic technologies | newborn screening; reproductive risk information; policy; ethics; informed decision making | Syst. review, global |
| Burke *et al*. (2010)(19) | Genetic technologies | Clinical utility; Evidence-based practice; Genetic testing | Review, NA |
| Cornejo *et al*. (2010)(20) | MS/MS | N/A | Review, Chile |
| Dhondt (2010)(21) | MS/MS and genetic technologies | N/A | Review, NA |
| Kasper *et al*. (2010)(22) | MS/MS | National Austrian Newborn Screening Program, tandem mass spectrometry, inherited metabolic disorders, inborn errors of metabolism. | Research, Austria |
| Fisher *et al*. (2011)(23) | MS/MS | Neonatal screening, Decision-making processes, Reimbursement, Health technology assessment, Fourth hurdle | Review, Europe |
| Hayeems *et al*. (2013)(24) | None | expanded newborn screening, mixed methods, public engagement, public expectations | Research, Canada |
| Bombard *et al*. (2014)(25) | Genetic technologies | N/A | Research, Canada |
| Botkin *et al*. (2014)(26) | None | newborn screening, research, research ethics, informed consent, parental permission | Meeting report, USA |

**Organizational abbreviations**: AAP: American Association of Pediatrics; ACMG: American College of Medical Geneticists; CADTH: Canadian Agency for Drugs and Technologies in Health; CCASS: Canadian Congenital Anomalies Surveillance System; CCMG: Canadian College of Medical Geneticists; CDCs: US Centers for Disease Control and Prevention; CORD: Canadian Organization for Rare Disorders; CPS: Canadian Paediatric Society; HRSA/MCHB: Health Resources and Services Administration/Maternal and Child Health Bureau; INTA: Institute of Nutrition and Food Technology; IOM: Institute of Medicine; NAS: National Academy of Sciences; NSC: UK National Screening Committee; PHAC: Public Health Agency of Canada; SACHDNC: Secretary’s Advisory Committee on Heritable Diseases in Newborns and Children; SOGC: Society of Obstetricians and Gynaecologists of Canada; WHO: World Health Organization.

**Other abbreviations**: CF: cystic fibrosis; DTC: direct-to-consumer; ELSIs: ethical, legal, and societal issues; HTA: health technology assessment; IDM: informed decision making; IEM: inborn errors of metabolism; MS/MS: tandem mass spectrometry; NBS: newborn bloodspot screening; NL: Netherlands UK: United Kingdom; USA: United States of America; WG/ES: whole genome/exome sequencing.

^a^ For example covering the following disciplines: expert on metabolic diseases, representative from the Newborn Screening Program, parent/consumer representative, researcher, representative of a Hospital Association, official from a Department of Public Health, medical ethicist, representative from public health laboratories; screening program administration; health care providers

**References**

1. Hiller EH, Landenburger G, Natowicz MR. Public Participation in Medical Policy-Making and the Status of Consumer Autonomy: The Example of Newborn Screening Programs in the United States. *American Journal of Public Health* (1997) **87**(8):1280-8.
2. Atkinson K, Zuckerman B, Sharfstein JM, Levin D, Blatt RJR, Koh HK. A Public Health Response to Emerging Technolgy: Expansion of the Massachusetts Newborn Screening Program. *Public Health Reports* (2001) **116**:122-31.
3. Therrell BL. U.S. Newborn Screening Policy Dilemmas for the Twenty-First Century. *Molecular Genetics and Metabolism* (2001) **74**:64-74.
4. Elliman DAC, Dezateux C, Bedford HE. Newborn and childhood screening programmes: criteria, evidence, and current policy. *Arch Dis Child* (2002) **87**:6-9.
5. Comeau AM, Parad R, Gerstle R, O'Sullivan BP, Dorkin HL, Dovey M, et al. Challenges in Implementing a Successful Newborn Cystic Fibrosis Screening Program. *J Pediatr* (2005) **147**:S89-S93.
6. Bailey DB, Beskow LM, Davis AM, Skinner D. Changing Perspectives on the Benefits of Newborn Screening. *MRDD Research Reviews* (2006) **12**:270-9.
7. Grosse SD, Boyle CA, Kenneson A, Khoury MJ, Wilfond BS. From Public Health Emergency to Public Health Service, The Implications of Evolving Criteria for Newborn Screening Panels. *Pediatrics* (2006) **117**(3):923-9.
8. Arn PH. Newborn Screening: Current Status. *Health Affairs* (2007) **26**(2):559-66.
9. Borowski HZ, Brehaut J, Hailey D. Linking evidence from health technology assessments to policy and decision making: The Alberta Model. *Intl J of Technology Assessment in Health Care* (2007) **23**(2):155-61.
10. Bailey DB, Skinner D, Davis AM, Withmarsh I, Powell C. Ethical, Legal, and Social Concerns About Expanded Newborn Screening: Fragile X Syndrome as a Prototype for Emerging Issues. *Pediatrics* (2008) **121**:e693-e704.
11. Little J, Potter B, Allanson J, Caulfield T, Carroll JC, Wilson B. Canada: Public Health Genomics. *Public Health Genomics* (2008) **12**:112-20.
12. Potter BK, Avard D, Entwistle V, Kennedy C, Chakraborty P, McGuire M, et al. Ethical, Legal, and Social Issues in Health Technology Assessment for Prenatal/Preconceptional and Newborn Screening: A Workshop Report. *Public Health Genomics* (2009) **12**:4-10.
13. Therrell BL. Considerations in Choosing Screening Conditions : One (US) Approach. *Ann Acad Med Singapore* (2008) **37**(Suppl 3):22-6.
14. Bailey DB. The Blurred Distinction between Treatable and Untreatable Conditions in Newborn Screening. *Health Matrix* (2009) **19**:141-53.
15. Plass AMC, van El CG, Pieters T, Cornel MC. Neonatal Screening for Treatable and Untreatable Disorders: Prospective Parents' Opinions. *Pediatrics* (2009) **125**:e99-e106.
16. Simopoulos AP. Genetic Screening: Programs, Priniciples, and Research - Thirthy Years Later. *Public Health Genomics* (2009) **12**:105-11.
17. Benkendorf J, Goodspeed T, Watson MS. Newborn screening residual dried blood spot use for newborn screening quality improvement. *Genet Med* (2010) **12**:-. doi: 10.1097.
18. Bombard Y, Miller FA, Hayeems RZ, Avard D, Knoppers BM. Reconsidering reproductive benefit through newborn screening: a systematic review of guidelines on preconception, prenatal and newborn screening. *European Journal of Human Genetics* (2010) **18**:751-60.
19. Burke W, Laberge A-M, Press N. Debating Clinical Utility. *Public Health Genomics* (2010) **13**:215-23.
20. Cornejo V, Raimann E, Cabello JF, Valiente A, Becerra C, Opazo M, et al. Past, present and future of newborn screening in Chile. *J Inherit Metab Dis* (2010) **33**(Suppl 3):S301-S6.
21. Dhondt J. Expanded newborn screening: social and ethical issues. *J Inherit Metab Dis* (2010) **33**(Suppl 2):S211-S7.
22. Kasper DC, Ratschmann R, Metz TF, Mechtler TP, Moslinger D, Konstantopoulou V, et al. The National Austrian Newborn Screening Program - Eight years experience with mass spectrometry. Past, present, and future goals. *Wien Klin Wochenschr* (2010) **122**:607-13.
23. Fischer KE, Grosse SD, Rogowski WH. The role of health technology assessment in coverage decisions on newborn screening. *Intl J of Technology Assessment in Health Care* (2011) **27**(4):313-21.
24. Hayeems RZ, Miller FA, Bombard Y, Avard D, Carroll JC, Wilson BJ, et al. Expectations and values about expanded newborn screening: a public engagement study. *Health Expectations* (2013).
25. Bombard Y, Miller FA, Hayeems RZ, Barg C, Cressman C, Carroll JC, et al. Public views on participating in newborn screening using genome sequencing. *Eur J Hum Genet* (2014) **22**(11):1248-54. doi: 10.1038/ejhg.2014.22.
26. Botkin JR, Lewis MH, Watson MS, Swoboda KJ, Anderson R, Berry SA, et al. Parental Permission for Pilot Newborn Screening Research: Guidelines From the NBSTRN. *Pediatrics* (2014) **133**:e410-e7
